# Effectiveness of multi-junction cells in near-field thermophotovoltaic devices considering additional losses

**DOI:** 10.1515/nanoph-2023-0572

**Published:** 2023-11-08

**Authors:** Jaeman Song, Minwoo Choi, Bong Jae Lee

**Affiliations:** Department of Mechanical Engineering, College of Engineering, Kyung Hee University, Yongin 17104, South Korea; Department of Mechanical Engineering, KAIST, 291, Daehak-ro, Yuseong-gu, Daejeon-si 34141, South Korea

**Keywords:** near-field radiative heat transfer, thermophotovoltaic device, series resistance losses, scalable design

## Abstract

Thermophotovoltaic (TPV) energy converters hold substantial potential in converting thermal radiation from high-temperature emitters into electrical energy through photovoltaic (PV) cells, offering applications ranging from solar energy harvesting to waste heat recovery. Near-field TPV (NF-TPV) devices, focused on enhancing power output density (POD), exhibit unique potential by harnessing photon tunneling. However, this potential can be mitigated by additional losses arising from high photocurrent densities and corresponding scalability issues. This study comprehensively investigates the effectiveness of multi-junction-based NF-TPV devices, accounting for additional losses. We propose two approximative expressions to quantify the impact of additional losses and characterize current density-voltage curves. Verification against rigorously optimized results establishes a criterion for effective performance. Our method provides precise POD estimations even for devices with 10 or more subcells, facilitating performance analysis across parameters like vacuum gap distance, cell width, emitter temperature, and the number of subcells compared to far-field counterparts. This research outlines a roadmap for the scalable design of NF-TPV devices, emphasizing the role of multi-junction PV cells. The analytical framework we developed will provide vital insights for future high-performance TPV devices.

## 1 Introduction

Thermophotovoltaic (TPV) energy converters transform thermal radiation emitted from high-temperature emitters into electrical energy through the photoelectric effect in photovoltaic (PV) cells [1]. These devices exhibit versatile functionality across various applications, operating as solar TPV systems with concentrated solar irradiation [2–4], contributing to industrial waste heat recovery [5, 6], and enabling the conversion of thermal energy stored in thermal batteries into electrical power [7]. Moreover, TPV converters integrate seamlessly with other high-temperature energy conversion technologies like solid-oxide fuel cells (SOFCs) [8], thermoelectric devices [9], and thermionic energy converters [10], forming hybrid systems. This inherent adaptability to various high-temperature environments of different scales underlines the versatility and scalability of TPV devices as energy conversion solutions.

Significant theoretical and experimental research has been dedicated to near-field TPV (NF-TPV) devices, focusing on enhancing power output density (POD) at specific emitter temperatures [11–14]. When the gap distance between the thermal emitter and PV cell becomes smaller than the thermal characteristic wavelength defined by Wien’s displacement law, evanescent waves can couple (i.e., photon tunneling), facilitating near-field radiation transfer [15–17]. Yet, maintaining vacuum gap distances at sub-micron scales poses considerable technical challenges. Nevertheless, the allure of NF-TPV devices lies in their potential to yield significantly amplified PODs, often surpassing far-field counterparts by several-fold to several-tens improvements.

To harness the energy generated by TPV devices, contacts must be placed on both the front and back surfaces of the PV cell. In this context, considering additional losses in performance analysis becomes essential that encompasses radiation absorption losses due to shading by the front contact grid (shading losses) and electrical losses from series resistance (series resistance losses) [18, 19]. Series resistance losses reduce the voltage at a given current density, and this reduction equals the product of the current density and the series resistance value. Consequently, the reduction is pronounced with increasing photocurrent densities and larger cell areas. As NF-TPV devices exploit enhanced photocurrent densities from near-field radiation, the enlargement of the cell area is limited by additional losses. While previous NF-TPV studies [18–20] have highlighted concerns about performance decline due to additional losses, these observations were confined to specific design conditions. A comprehensive analysis addressing the performance degradation caused by additional losses in the NF-TPV devices is notably absent.

To address the additional losses and enhance conversion efficiency in NF-TPV devices, several studies have investigated the use of multi-junction PV cells [20–23]. These cells feature subcells organized in a sequence where the bandgap energy decreases from the emitter side to the back contact, ensuring efficient spectral absorption. The performance of multi-junction-based NF-TPV devices must be evaluated with cell configurations that meet the current matching condition. However, achieving the current matching condition at a specific design configuration requires an intricate optimization process, which hindered the comprehensive analysis under diverse design situations [22]. Hence, streamlining the evaluation of multi-junction-based NF-TPV device performance, while accounting for additional losses, will greatly facilitate the design of practical NF-TPV devices.

This study presents a comprehensive exploration into the effectiveness of multi-junction-based NF-TPV energy conversion devices, considering the effect of additional losses. Two approximative expressions are introduced to evaluate the device performance much more efficiently but accurately: the first quantifies the effect of additional losses, and the second gives the current density-voltage (*J*-*V*) characteristics curve of multi-junction cells. We have devised an expression for a factor that characterizes performance degradation arising from additional losses, which can be applied to multi-junction-based TPV devices. By validating the approximations against rigorous calculations, we establish design criteria for achieving effective performance. The introduced approximation method evaluates the performance of multi-junction-based NF-TPV devices in line with current matching conditions, facilitating precise predictions and eliminating the need for structure optimization of PV cells. By combining the developed methodologies, the degree of performance enhancement in NF-TPV devices relative to far-field counterparts is systematically explored across design conditions encompassing vacuum gap distance, cell width, emitter temperature, and the number of subcells.

## 2 Theory and method

**Figure 1:**
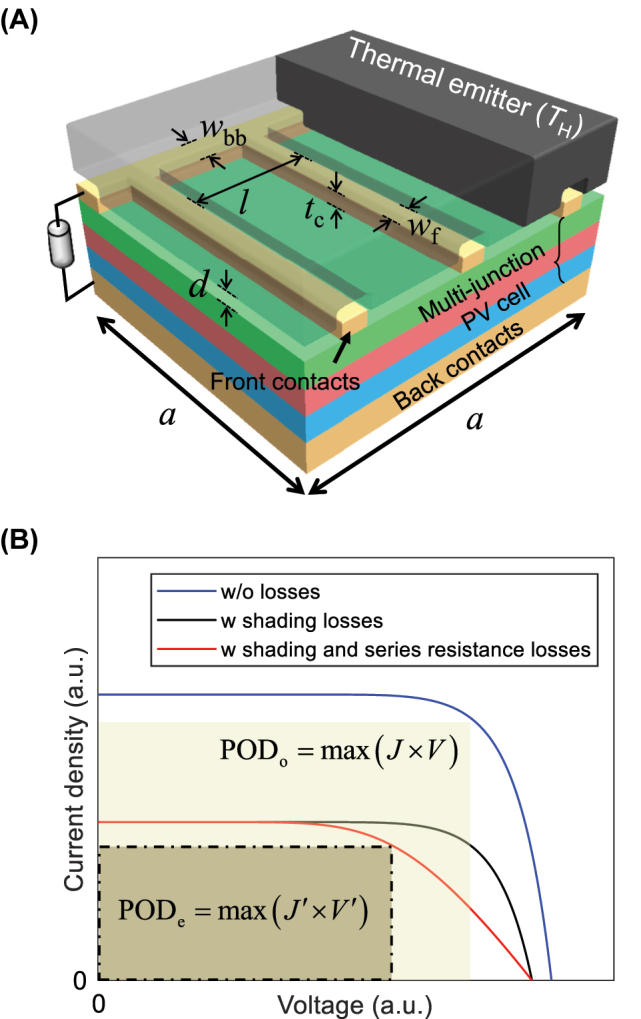
Multi-junction-based near-field thermophotovoltaic (NF-TPV) devices under investigation. (A) Schematic illustration of NF-TPV devices. (B) Current density-voltage characteristic curves.

TPV devices under investigation consist of a bulk graphite emitter at a temperature *T*_H_ and a PV cell with dimensions *A*_cell_ = *a* × *a* and a temperature of 300 K, as depicted in Figure 1(A). To facilitate the transfer of near-field thermal radiation, the distance *d* between the emitter and the PV cell is maintained at levels smaller than the thermal characteristic wavelength. The subcells constituting the multi-junction PV cell are arranged in decreasing order of bandgap, with the top cell geared to absorb short-wavelength radiation. The absorption is sequentially followed by the middle and bottom cells. Each subcell is formed by Ga_*x*_In_1−*x*_As_*y*_Sb_1−*y*_ quaternary semiconductor, ensuring lattice constant matches with GaAs within a 2 % tolerance. Electrical properties (e.g., bandgap energy, carrier mobility, and carrier lifetime) and optical properties (e.g., real and imaginary components of permittivity) can be determined based on the composition (*x*, *y*) using Vegard’s law [24, 25].

The Au back contact is positioned at the base of the thin film PV cell, which collects photocurrent while reflecting the sub-bandgap radiative energy that is transmitted through the PV cell back towards the emitter to minimize photonic loss [26]. On the top surface of the PV cell, the Au front contact grid composed of a busbar and an array of fingers is patterned to collect photocurrent. Its dimensional parameters are: finger width *w*_f_, period between fingers *l*, and busbar width *w*_bb_. *l* exceeds 40 μm to mitigate potential interference effects between contact fingers while *w*_f_ ≥ 20 μm. The thickness of the front contact *t*_c_ is set to be 200 nm based on insights from prior research [19]. The magnitude of series resistance losses associated with the dimensional parameters is calculated based on analytic relations in [19]. In each design scenario introduced in Section 3, *w*_f_, *l*, and *w*_bb_ are systematically optimized through a genetic algorithm so that the power output density (POD) is maximized regarding the additional losses including the series resistance and shading losses. In cases where the vacuum gap distance becomes smaller than *t*_c_, the emitter surface should be grooved to align with the shape of the front contact grid to prevent contact with the PV cell. While thinner front contacts have the potential to simplify the design of NF-TPV devices, they may not effectively mitigate series resistance, leading to substantial performance losses. The quantitative impact of *t*_c_ on the performance of NF-TPV devices is described in Figure S1 of the Supplementary Material.

### 2.1 Approximate expression of additional losses factor in TPV devices

The performance of the PV-based energy conversion devices can be evaluated through the *J*-*V* characteristic curve, illustrated in Figure 1(B). Based on the analytical approximation model [14], the *J*-*V* equation, which excludes the consideration of additional losses, can be formulated as follows:

(1) $$J\left(V\right)=J_{\text{sc}}-J_{0}\left\{exp\left(\frac{qV}{k_{\text{B}}T_{\text{L}}}\right)-1\right\}$$

where *J*_sc_ is the short-circuit current density, *q* is the electron volt, *k*_B_ is Boltzmann constant, and *T*_L_ is the temperature of the PV cell. $J_{0}=q\left(\frac{n_{i}^{2}}{N_{A}}\sqrt{\frac{D_{n}}{\tau_{n}}}+\frac{n_{i}^{2}}{N_{D}}\sqrt{\frac{D_{p}}{\tau_{p}}}\right)$ is the saturation current density [16, 19] where *D*_*n*,*p*_ and *τ*_*n*,*p*_ are the diffusion coefficient and lifetime of minority carriers in *p* or *n* regions, respectively, and *n*_*i*_, *N*_*A*_, and *N*_*D*_ are the concentration of intrinsic carriers, acceptors, and donors, respectively. The total minority carrier lifetimes are determined using Matthiessen’s rule as $\tau^{-1}=\tau_{\text{rad}}^{-1}+\tau_{\text{SRH}}^{-1}+\tau_{\text{Auger}}^{-1}$ . It includes the radiative recombination lifetime *τ*_rad_, nonradiative Shockley–Read–Hall (SRH) recombination lifetime *τ*_SRH_, and Auger recombination lifetime *τ*_Auger_. The voltage value corresponding to *J* being 0 is referred to as the open-circuit voltage (*V*_oc_). The maximum POD is determined as the multiplication of the current density and voltage having the maximum attainable value. Specifically, POD_o_ is computed as *FF*_o_ *J*_sc_ *V*_oc,o_, where *FF*_o_ represents the fill factor, and the subscript “o” signifies the scenario without factoring in additional losses. With an increase in the absorption of photons having larger energy than the bandgap of the PV cell, *J*_sc_ experiences growth, leading to the generation of a higher POD. In the presence of shading losses attributed to the front contact grid, a modified *J*′-*V* curve can be applied as follows:

(2) $$J^{\prime }\left(V\right)=J_{\text{sc}}\left(1-F_{s}\right)-J_{0}\left\{exp\left(\frac{qV}{k_{\text{B}}T_{\text{L}}}\right)-1\right\}$$

where *F*_*s*_ is the shading fraction. As depicted in Figure 1(B), the incorporation of shading losses results in a reduction in current density by a factor of (1 − *F*_*s*_). To account for various resistances, including sheet resistance, finger resistance, busbar resistance, and substrate resistance, a normalized series resistance *r*_*s*_ = *R*_*s*_ *A*_cell_ where *R*_*s*_ is the series resistance, is introduced. Due to the presence of series resistance losses, there is a corresponding reduction in voltage, leading to the contraction of the *J*′-*V* curve’s area. The *J*′-*V*′ curve, which additionally represents the impact of series resistance losses, can be expressed in the following manner:

(3) $$J^{\prime }\left(V^{\prime }\right)=J_{\text{sc}}\left(1-F_{s}\right)-J_{0}\left\{exp\left(\frac{qV^{\prime }}{k_{\text{B}}T_{\text{L}}}\right)-1\right\}$$

where *V*′ = *V* − *J*′*r*_*s*_. The power output density impacted by additional losses is denoted as POD_e_ = *FF*_e_(1 − *F*_*s*_)*J*_sc_ *V*_oc,e_, with the subscript “e” indicating the consideration of additional losses. In general, it has been well-established that NF-TPV devices can yield significantly heightened POD_o_ due to the near-field radiation effect, driven by the contribution of evanescent waves. However, with an increase in current density facilitated by this effect, there is a corresponding augmentation in voltage reduction attributed to *r*_*s*_, particularly when *A*_cell_ is large. Consequently, the anticipated performance enhancement might not be fully realized. To quantitatively gauge the extent of performance reduction, we introduce an additional losses factor *α* that adheres to the equation POD_e_ = *α*POD_o_. This approach offers the means to comprehend the degree of performance diminution and formulate strategies to mitigate it.

Under moderate *r*_*s*_, it is reasonable to posit that the current density at which the power output density becomes maximum remains relatively unaffected by series resistance losses. Furthermore, assuming that shading losses exert minimal impact on the fill factor (validated in Figure S2 of the Supplementary Material), the expression for POD_e_ can be presented in the following manner:

(4) $$\begin{array}{cc} {\text{POD}}_{\text{e}} & \approx V_{M}J_{M}^{\prime }-{J_{M}^{\prime }}^{2}r_{s}\\ & \approx FF_{\text{o}}\left(1-F_{s}\right)J_{\text{sc}}V_{\text{oc,e}}\left\{1-\frac{J_{\text{sc}}\left(1-F_{s}\right)r_{s}}{V_{\text{oc,e}}}\right\}\\ & =\left(1-F_{s}\right)\gamma \frac{V_{\text{oc,e}}}{V_{\text{oc,o}}}{\text{POD}}_{\text{o}} \end{array}$$

where *γ* = 1 − *J*_sc_(1 − *F*_*s*_)*r*_*s*_/*V*_oc,e_. *V*_*M*_ and $J_{M}^{\prime }$ are the voltage and current density that correspond to the maximum power output density, respectively. When the second line of Eq. (4) is derived from the first line, it should be assumed that $J_{M}^{\prime }/V_{M}\approx J_{\text{sc}}\left(1-F_{s}\right)/V_{\text{oc,e}}$ , and this is validated in Figure S3 of the Supplementary Material. Consequently, we are able to express $\alpha =\left(1-F_{s}\right)\gamma V_{\text{oc,e}}/V_{\text{oc,o}}$ , leading to the realization that, for a given *r*_*s*_ and *F*_*s*_, maximizing *α* necessitates a reduction in *J*_sc_ coupled with an elevation in *V*_oc,e_. It is foreseeable that the introduction of a multi-junction PV cell into TPV devices can serve as an effective means of alleviating additional losses. The subcells within the multi-junction PV cell can separately convert thermal radiative energy into electrical energy (leading to the reduction of *J*_sc_), with each subcell serially interconnected (leading to the elevation of *V*_oc_). With consideration of the number of subcells *n* comprising the multi-junction PV cell, the definitions for *V*_oc,o_, *V*_oc,e_, and *FF*_o_ can be written as follows:

(5) $$V_{\text{oc,o}}=\frac{k_{\text{B}}T_{\text{L}}}{q}\left\{\overset{n}{\underset{k=1}{\sum }}ln\left(\frac{J_{\text{sc},k}}{J_{0,k}}+1\right)\right\}$$

(6) $$V_{\text{oc,e}}=\frac{k_{\text{B}}T_{\text{L}}}{q}\left[\overset{n}{\underset{k=1}{\sum }}ln\left\{\frac{J_{\text{sc},k}\left(1-F_{s}\right)}{J_{0,k}}+1\right\}\right]$$

(7) $$FF_{\text{o}}=\left(1-\frac{1}{y}\right)\left(1-\frac{\text{ln}y}{y}\right)$$

where $y=\ln\left(J_{\text{sc}}/J_{\text{0,eff}}\right)$ with *J*_sc_ being the minimum short-circuit current density among the subcells, and *J*_0,eff_ signifying the effective saturation current density, approximately derived through the harmonic mean of all saturation current densities obtained by the electrical characteristics of each subcell. By employing Eqs. (4)–(7) in conjunction with the definition of POD_o_ = *FF*_o_ *J*_sc_ *V*_oc,o_, both *α* and POD_e_ can be readily computed for specified *J*_sc_, *F*_*s*_, and *r*_*s*_, irrespective of the number of subcells present in the multi-junction PV cell.

### 2.2 Approximate expression of *J*-*V* characteristics in multi-junction NF-TPV devices

To analyze the impact of additional losses, it is required to find the *J*-*V* characteristic (i.e., *J*_sc_ and *J*_0_) of multi-junction NF-TPV devices in the absence of these losses. In any design configuration, *J*_sc_ can be obtained as the minimum short-circuit current density among the subcells. However, excess photocurrent in a specific subcell can induce thermalization loss, which prohibits analysis of the effectiveness of the multi-junction cells. One method to resolve the issue is finding design configurations that meet the current matching condition and deriving their performance. This means designing different multi-junction PV cells for each combination of the temperature of an emitter *T*_H_ and the vacuum gap distance *d*. The design is accomplished by finding the combination of variables, taking each subcell’s composition and thickness into account, that maximizes the conversion efficiency [20]. The simulation for calculating conversion efficiency encompasses a layer-by-layer absorption considering both forward and backward waves in each layer [16, 27]. Therefore, the computational load can make the optimization infeasible, especially when the number of subcells *n* increases. To analyze the effect of additional losses at various ranges of *T*_H_, *d*, and *n*, we propose an approximation method that concisely estimates *J*_sc_ and *J*_0_ of current-matched multi-junction NF-TPV devices.

For demonstration, we find approximate values of *J*_sc_ and *J*_0_ for a triple-junction-based NF-TPV device operating at *T*_H_ = 1500 K and *d* = 100 nm. The initial step is the determination of the frequency corresponding to the bandgap energy of the bottom cell *ω*_*g*,1_. Subsequently, the computation entails the evaluation of the near-field thermal radiative heat flux between the single-junction GaInAsSb PV cell inclusive of the back contact, and the graphite emitter. This calculation is conducted utilizing the following formula:

(8) $$\begin{array}{cc} Q & =\overset{\infty }{\underset{0}{\int }}d\omega \;Q_{\omega }=\overset{\infty }{\underset{0}{\int }}d\omega \;\left(Q_{\text{prop},\omega }+Q_{\text{evan},\omega }\right)\\ & =\underset{j=p,s}{\sum }\overset{\infty }{\underset{0}{\int }}d\omega \;\frac{\Theta \left(\omega ,T_{\text{H}}\right)-\Theta \left(\omega ,T_{\text{L}}\right)}{4\pi^{2}}\\ & \;\times \left\{\overset{\omega /c_{0}}{\underset{0}{\int }}Z_{\text{prop},\beta ,\omega }^{j}\left(\beta ,\omega \right)d\beta +\overset{\infty }{\underset{\omega /c_{0}}{\int }}Z_{\text{evan},\beta ,\omega }^{j}\left(\beta ,\omega \right)d\beta \right\} \end{array}$$

where $\Theta \left(\omega ,T\right)=\hbar \omega /\left\{\exp\left[\hbar \omega /\left(k_{B}T\right)\right]-1\right\}$ is the mean energy of the Planck oscillator with *ℏ* being the reduced Planck constant. The exchange functions for propagating and evanescent waves can be expressed as:

(9) $$\begin{array}{cc} & Z_{\text{prop},\beta ,\omega }^{p,s}\left(\beta ,\omega \right)=\frac{\beta \left(1-|r_{01}^{p,s}{|}^{2}\right)\left(1-|r_{02}^{p,s}{|}^{2}\right)}{|1-r_{01}^{p,s}r_{02}^{p,s}e^{i2k_{0z}d}{|}^{2}}\\ & Z_{\text{evan},\beta ,\omega }^{p,s}\left(\beta ,\omega \right)=\frac{4\beta \text{Im}\left(r_{01}^{p,s}\right)\text{Im}\left(r_{02}^{p,s}\right)e^{-2\text{Im}\left(k_{0z}\right)d}}{|1-r_{01}^{p,s}r_{02}^{p,s}e^{i2k_{0z}d}{|}^{2}} \end{array}$$

where $r_{01}^{p,s}$ and $r_{02}^{p,s}$ are the modified reflection coefficients, determined by Airy’s formula [28, 29], for the emitter side and PV cell side, respectively. *k*_0*z*_ is the normal component of the wavevector in vacuum and Im() takes the imaginary part of a complex value. The permittivity of the materials composing the NF-TPV devices can be obtained from tabulated data for graphite [30], the Drude model for Au [31], and the semi-empirical model suggested by Adachi [32] together with Drude model, both assisted by Vegard’s law in the case of the PV cell [25]. To ensure the sufficient absorption of photon energy exceeding the bandgap energy, the thickness of the PV cell was established as 600 nm. Assuming an internal quantum efficiency of 100 % which is common for thin-film PV cells, the total photocurrent density can be calculated as follows:

(10) $$J_{\text{ph}}=\overset{\infty }{\underset{\omega_{g}}{\int }}d\omega \;J_{\text{ph},\omega }=\overset{\infty }{\underset{\omega_{g}}{\int }}d\omega \frac{q}{\hbar \omega }Q_{\omega }$$

In multi-junction-based NF-TPV devices, the optimal conversion efficiency is achieved when the current-matching condition is fulfilled where the generated photocurrent densities at all subcells are the same. Therefore, we assume the current matching condition in a multi-junction-based NF-TPV device with *n* subcells, so that the short-circuit current density of the *k-*th (i.e., 1 ≤ *k* ≤ *n*) subcell can be simply given as *J*_sc,*k*_ = *J*_ph_/*n*. Presuming each subcell neither transmits in the above-bandgap region nor absorbs in the sub-bandgap region, the frequencies corresponding to the bandgap energies for subsequent subcells, *ω*_*g*,2_ and *ω*_*g*,3_, can be sequentially determined. This principle is succinctly captured in the following equations:

(11) $$J_{\text{sc},k}=\overset{\omega_{g,k+1}}{\underset{\omega_{g,k}}{\int }}d\omega \frac{q}{\hbar \omega }Q_{\omega }\;\;\text{for}\;1\leq k\leq n-1$$

(12) $$J_{\text{sc},n}=\overset{\infty }{\underset{\omega_{g,n}}{\int }}d\omega \frac{q}{\hbar \omega }Q_{\omega }$$

where *J*_sc,*k*_ have same magnitude each other.

**Figure 2:**
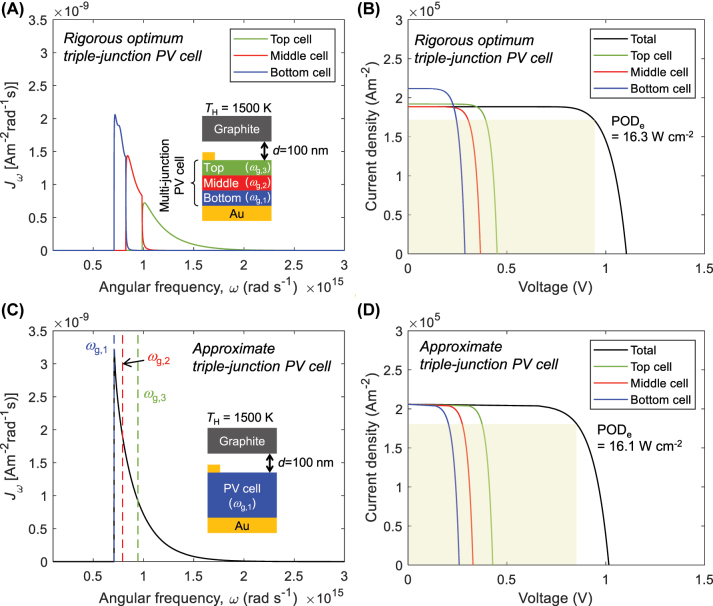
Comparative analysis between rigorous-optimum and approximate computations of the triple-junction-based near-field thermophotovoltaic (NF-TPV) device. (A) Spectral photocurrent density generated in the top, middle, and bottom cells of triple-junction PV cell optimized for graphite emitter at 1500 K and *d* = 100 nm. (B) *J*-*V* characteristic curves of the optimum PV cell in (A). (C) Spectral photocurrent density generated in the single-junction PV cell, where *ω*_*g*,2_ and *ω*_*g*,3_ values that satisfy the current matching condition are depicted. They determine the approximate configuration of the triple-junction PV cell. (D) *J*-*V* characteristic curves of the approximate PV cell determined in (C).

Figure 2 provides a comparative analysis between rigorous-optimum and approximate computations for the spectral photocurrent density (*J*_ph,*ω*_) and *J*-*V* characteristic curves of the triple-junction-based NF-TPV device. The structural configuration corresponding to rigorous-optimal outcomes follows that presented in a previous study [22], which is rigorously computed by a multi-junction-compatible minority carrier separation (MCS) model [20] and optimized through the integration of an ANN-based surrogate model and a genetic algorithm [22]. In Figure 2(A), it can be seen that the bandgap energies of the top, middle, and bottom subcells were determined to fulfill the current matching condition. The corresponding POD_e_ was 16.3 W cm^−2^ (see Figure 2(B)). Figure 2(C) illustrates the positions of *ω*_*g*,2_ and *ω*_*g*,3_, approximately determined based on the outcomes of the single-junction PV cell with *ω*_*g*,1_ and Eqs. (11) and (12). The *J*-*V* curve resulting from this approximation method is displayed in Figure 2(D), with a corresponding POD_e_ of 16.1 W cm^−2^. In comparison to the rigorous calculation, a substantial level of accuracy is ascertained, as the approximation exhibits only a 1.2 % error.

The *J*-*V* characteristics approximation method outlined here comes with certain limitations. Notably, it is not capable of calculating the absorption spectrum in each layer of PV cells independently. In accordance, absorption losses arising from factors like back contacts and tunnel diodes cannot be calculated. Additionally, the method has challenges in accounting for absorption enhancements triggered by resonance modes such as surface plasmon polaritons. It is applicable when adopting bulk isotropic medium, such as graphite, as an emitter. Nevertheless, given the inherent fabrication complexities associated with various resonance modes, surface modifications may hinder the maintenance of sub-micron gaps which stands as a paramount consideration in NF-TPV devices. Therefore, our approximation method will offer valuable assistance in the prompt design of multi-junction-based NF-TPV devices.

Regarding the performance analysis model, we have employed the analytical approximation model. This choice is grounded in several key considerations specific to our study: the gap size is consistently maintained above 100 nm, the doping concentration of the semiconductor is moderate (1e17 cm^−3^), thin-film PV cells are employed, and the emitter material is bulk graphite. Due to these factors, approximation model can ensure a sufficiently accurate performance analysis of our NF-TPV devices. However, it is important to note that if the specific NF-TPV device cannot be effectively simulated using the analytical approximation model (for instance, in scenarios where the PV cell structure necessitates the consideration of internal quantum efficiency (IQE), an emitter that supports surface mode is utilized, or the gap size is extremely small on the order of 10 nm), the analysis should be conducted based on an alternative performance model. In such cases, models such as the MCS model or the Poisson drift-diffusion (PDD) model would be more appropriate to the accurate analysis of device performance [14].

**Figure 3:**
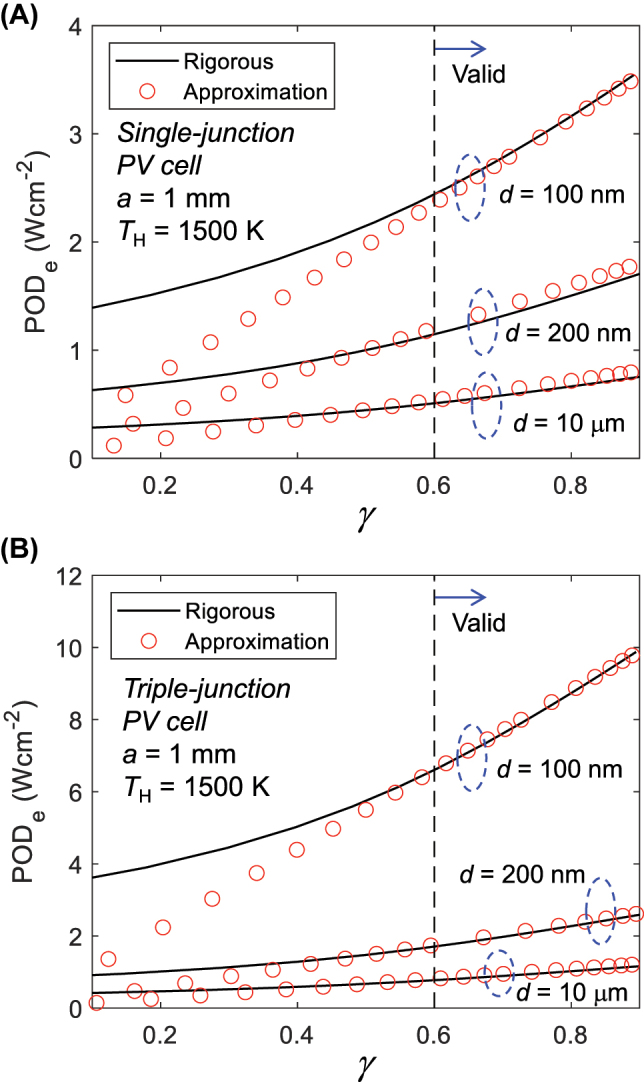
Validation of approximative expressions for predicting the performance of multi-junction-based near-field thermophotovoltaic (NF-TPV) devices. (A) POD_e_ of TPV device utilizing single-junction PV cell as a function of *γ*. Results obtained with the rigorous and approximative expressions are compared in the near-field (*d* = 100 and 200 nm) and far-field (*d* = 10 μm) regimes. (B) The same analysis as in (A), but with the devices that utilize a triple-junction PV cell.

Figure 3 illustrates approximated POD_e_ derived using the method outlined in subsections 2.1 and 2.2 together with rigorously calculated values, plotted as a function of *γ*. As introduced in Ref. [20], rigorous values are derived through computations using a multi-junction-compatible MCS model and optimization via a genetic algorithm. The design of the front contacts remains consistent for both rigorous and approximated simulations, with only *t*_c_ being appropriately adjusted to observe POD_e_ relative to *γ*. Figure 3(A) and (B) show results for TPV devices equipped with single-junction and triple-junction PV cells, respectively, for gap distances *d* of 100 nm, 200 nm and 10 μm. Irrespective of the gap distance and the number of subcells, a notable observation emerges: rigorous and approximated POD_e_ align closely only when *γ* exceeds 0.6. Stated differently, when *γ* falls below 0.6, it implies that the configuration of *J*-*V* curve becomes overly constrained due to series resistance. This criterion can serve as a pivotal factor in evaluating the effectiveness of the designed NF-TPV devices. Further elaboration on the validity range of *γ* > 0.6 for our approximative expression is outlined in Figure S4 of the Supplementary Material. Additionally, supplementary validations of the approximation of POD_e_ across various emitter temperatures and gap sizes are presented in Figure S5 of the Supplementary Material.

## 3 Results and discussion

Theoretical investigations into NF-TPV devices have predominantly focused on enhancing the performance of POD_o_ without accounting for additional losses. However, in the practical design of TPV devices, factors such as the unit cell size and the configuration of front contact grid must be reflected, requiring the consideration of additional losses. In this context, the utilization of approximation methods for obtaining the factor of additional losses and *J*-*V* characteristics, as introduced in Section 2, offers a means to comprehensively evaluate the performance of multi-junction-based NF-TPV devices across varying parameters such as vacuum gap distance and emitter temperature. This approach aids in establishing initial design conditions. This section encompasses two explorations concerning multi-junction-based NF-TPV devices. Firstly, for a given emitter temperature, we delve into the extent to which performance enhancements, relative to the far-field counterpart, can be achieved by adjusting the vacuum gap distance and cell width. Subsequently, with fixed vacuum gap distance and cell width, we analyze power generation as a function of emitter temperature and analyze how the number of subcells influences device performance.

**Figure 4:**
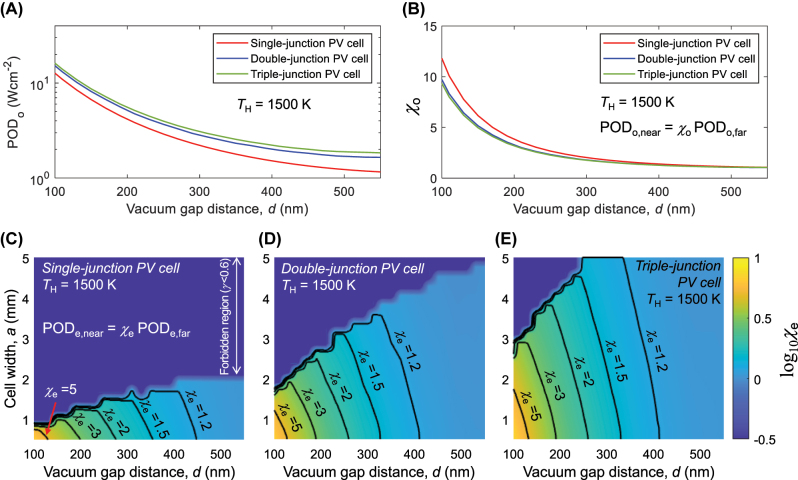
Enhancement factors of multi-junction-based near-field thermophotovoltaic (NF-TPV) devices with respect to the cell width and vacuum gap distance. (A) POD_o_ of NF-TPV devices with different numbers of subcells as a function vacuum gap distance, where additional losses are neglected. (B) The enhancement factor for POD_o_ shown in (A), relative to far-field cases. (C)–(E) The enhancement factor for POD_e_ with consideration of additional losses as a function of cell width and vacuum gap distance, calculated with (C) single-junction, (D) double-junction, and (E) triple-junction PV cells.

A critical aspect of NF-TPV device research centers on determining the potential amplification of POD when compared to far-field devices under specified design parameters. We quantify the relative enhancement with enhancement factors *χ*_o_ and *χ*_e_ corresponding to POD_o_ and POD_e_, respectively. Figure 4 shows the POD and enhancement factors of multi-junction-based NF-TPV devices under an emitter temperature of 1500 K. In Figure 4(A), the range of POD_o_ (i.e., without considering additional losses) is depicted for vacuum gap distances spanning 100–550 nm. Remarkably, a POD_o_ of larger than 10 W cm^−2^ can be generated when the gap distance is 100 nm, regardless of the number of subcells. As the number of subcells rises, the magnitude of POD_o_ is enhanced at the entire gap distance range. This phenomenon is explainable by the incrementally increasing *V*_oc_ in the sequence of bottom, middle, and top cells, as displayed in Figure 2(B) or (D). Given that the frequency corresponding to the bandgap energy of the bottom cell (*ω*_*g*,1_) remains constant, and the larger bandgap subcells are progressively stacked on it, the dark current can be reduced with larger subcell numbers, leading to a boost in the size of POD. In Figure 4(B), the enhancement factor *χ*_o_ = POD_o,near_/POD_o,far_ is depicted with respect to the vacuum gap distance. The far-field reference is based on outcomes with *d* = 10 μm. POD_o_ is improved by a factor of around 10 when the vacuum gap distance is 100 nm.

The enhancement factor also becomes a function of the cell width by incorporating additional losses into the design. Figure 4(C)–(E) illustrate the enhancement factor *χ*_e_ = POD_e,near_/POD_e,far_ calculated as a function of vacuum gap distances and cell widths for single-, double-, and triple-junction-based NF-TPV devices. For every design point depicted in Figure 4(C)–(E), the front contact grid’s dimensional parameters are adjusted to maximize POD_e_. The blue-colored forbidden region represents conditions where effective power generation is challenging due to *γ* falling below 0.6 as a result of excessive series resistance losses. Notably, even at the same vacuum gap distances, *χ*_e_ diminishes as cell width increases. For the single-junction PV cell case, it is advisable to avoid cell widths exceeding 2 mm (see Figure 4(C)). As the number of junctions increases, accommodation of larger cell widths in NF-TPV configurations becomes a reasonable choice. In scenarios where *d* = 100 nm and *a* = 2 mm, *χ*_e_ exceeding 4.5 is attainable as shown in Figure 4(E). However, even with the introduction of a triple-junction PV cell, it remains intricate to construct an effective NF-TPV device under the conditions of *T*_H_ = 1500 K, *d* = 100 nm, and *a* = 3 mm. Consequently, designing an effective NF-TPV device under elevated temperatures, broader areas, and narrower vacuum gap distances is facilitated by further increasing the number of subcells.

**Figure 5:**
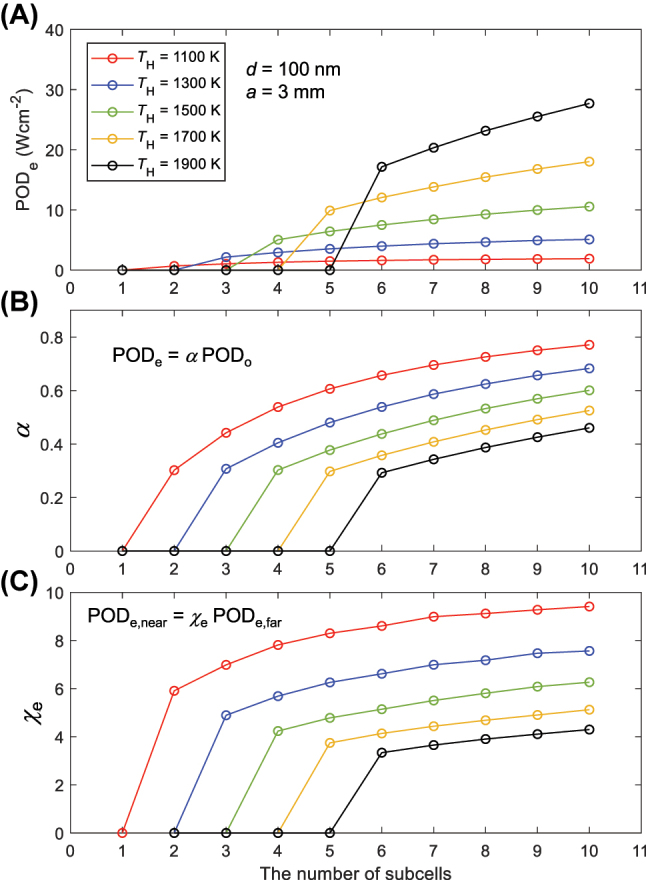
Enhancement factors of multi-junction-based near-field thermophotovoltaic (NF-TPV) devices with respect to the number of subcells and emitter temperature. (A) POD_e_ of NF-TPV devices with different numbers of subcells at various emitter temperatures. (B) The additional losses factor calculated for the NF-TPV devices in (A). (C) The enhancement factor for POD_e_ calculated for the NF-TPV devices in (A).

Figure 5 shows the number of subcells required to ensure the effective performance of multi-junction-based NF-TPV devices at different emitter temperatures. When the vacuum gap distance is fixed at 100 nm and the cell width is 3 mm, POD_e_, *α*, and *χ*_e_ are calculated for different numbers of subcells while increasing the temperature of the emitter from 1100 to 1900 K. In Figure 5(A), POD_e_ is shown as 0 under the condition that the design criterion *γ* is less than 0.6. As mentioned in Figure 4(E), and also repeated in Figure 5, it is difficult to expect effective performance from a triple-junction-based NF-TPV device under the conditions of *T*_H_ = 1500 K, *d* = 100 nm, and *a* = 3 mm. Effective performance can be guaranteed only when the number of subcells is increased to at least four, and as the temperature increases, more subcells are required. Additional losses factor *α* increase as the number of subcells increases and decreases as the temperature of the emitter increases (see Figure 5(B)). When the emitter temperature is 1900 K, *α* falls below 0.5 even with up to 10 subcells, implying that the power reduction by additional losses exceeds the generated power output. This illustrates the challenges in designing scalable NF-TPV devices that can operate effectively at elevated temperatures. Finally, the relative performance enhancement of NF-TPV devices compared to far-field devices is shown in Figure 5(C). Even when the emitter temperature is relatively low at 1100 K, a double-junction PV cell is required to attain effective performance, which exhibits a 6-fold enhancement compared to its far-field counterpart. Moreover, designing for emitters with temperatures of 1700 K or above precludes the possibility of attaining a *χ*_e_ > 6, regardless of the number of subcells used.

## 4 Conclusions

NF-TPV devices present significant potential as energy converters capable of enhancing power output density (POD) by harnessing the additional evanescent waves to thermal radiation. Nevertheless, the escalation of additional losses due to increased photocurrent in the PV cell has emphasized the need for estimating true performance gain. We systematically investigated the effectiveness of multi-junction-based NF-TPV devices, while accounting for size-dependent additional losses. A factor *α* was introduced to describe the performance decrement of TPV devices due to additional losses. This metric revealed that reducing additional losses could be effectively achieved by decreasing *J*_sc_ while augmenting *V*_oc_, prompting employment of multi-junction PV cells as a viable solution. Also, a design criterion for NF-TPV device to achieve effective performance was established as *γ* = 1 − *J*_sc_(1 − *F*_*s*_)*r*_*s*_/*V*_oc,e_ > 0.6.

Furthermore, we developed a technique to estimate the *J*-*V* characteristic curve of multi-junction NF-TPV devices with optimized subcell structures. By presuming the condition of current matching, we avoided the exhaustive calculations needed for rigorous optimization. This allowed for a swift derivation of POD for devices with 10 or more subcells with an acceptable accuracy level. Using the method, the relative enhancement in POD compared to the far-field devices was systematically explored through two distinct analyses: (1) concerning the gap distance and cell width, and (2) concerning the number of subcells and emitter temperature. It not only offered quantitative guidance for the construction of large-scale NF-TPV devices but also outlined a comprehensive roadmap for future endeavors. Furthermore, the analytical approach presented in this study offers crucial insights for designing scalable NF-TPV devices, emphasizing the role of multi-junction PV cells.

## Supplementary Material

Supplementary Material Details
