# Supplementary material for: Effectiveness of multi-junction cells in near-field thermophotovoltaic devices considering additional losses
Source: Nanophotonics. 2023 Nov 8;13(5):813–23. doi: 10.1515/nanoph-2023-0572 (PMC11502035; doi:10.1515/nanoph-2023-0572)
Supplement: Supplementary file 1 — Supplementary Material Details [file j_nanoph-2023-0572_suppl_001.pdf]

Supplementary Material:

Effectiveness of multi-junction cells in near-field thermophotovoltaic devices

Authors: Jaeman Song[1], Minwoo Choi[2], Bong Jae Lee[2*]

1. Department of Mechanical Engineering, College of Engineering, Kyung Hee University, Yongin 17104, South Korea

2. Department of Mechanical Engineering, KAIST, 291, Daehak-ro, Yuseong-gu, Daejeon-si 34141, South Korea

*Corresponding author

*e-mail: bongjae.lee@kaist.ac.kr (Bong Jae Lee)

Table of Contents

Supplementary Figure S1: The effect of the front contact thickness t_{c} on the performance degradation factor γ

Supplementary Figure S2: The effect of the shading fraction F_s on the fill factor FF.

Supplementary Figure S3: Similarity of values between J'_M/V_M and $J_{\mathrm{sc}}/V_{\mathrm{oc}}$.

Supplementary Figure S4: Establishment of threshold criterion for the validity of the approximative expression.

Supplementary Figure S5: Effectiveness of the threshold criterion over various design parameter ranges.

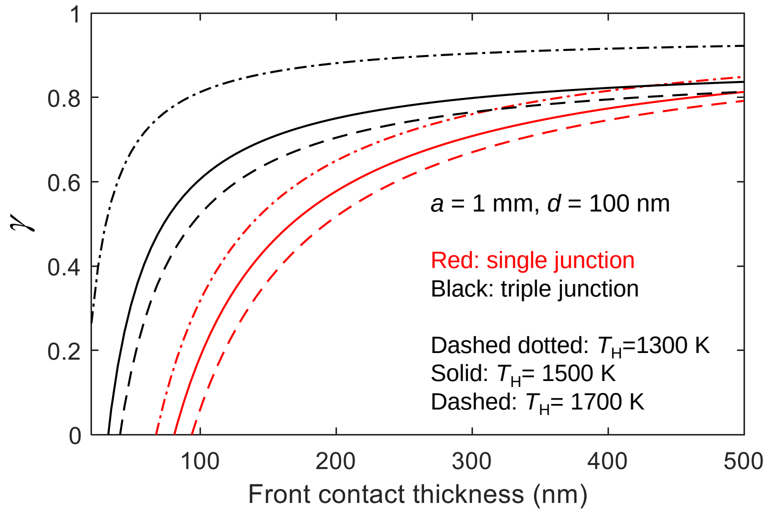

Fig. S1: Front contacts thinner than the vacuum gap size can simplify the emitter design, but can lead to substantial performance losses due to increased additional losses. The relationship between the performance degradation factor by additional losses, γ, and the front contact thickness t_c is presented for devices utilizing single-junction and triple-junction PV cells at three different emitter temperatures. At a vacuum gap of 100 nm and device area of 1 $\times$ 1 mm^2, the implementation of efficient NF-TPV devices becomes challenging when t_c $\leq$ 100 nm, particularly in the case of single-junction PV cells, which fail to meet the $\gamma > 0.6$ criterion essential for scalability. Even with the incorporation of triple-junction PV cells, elevating the emitter temperature over 1500 K proves to be a difficult task when the front contact is thinner than the vacuum gap size. Although the minimum required t_c varies based on several design parameters, a front contact thicker than the vacuum gap is essential for effective and scalable design in many instances. It can be clearly seen that a larger t_c facilitates more straightforward scalability in NF-TPV device design.

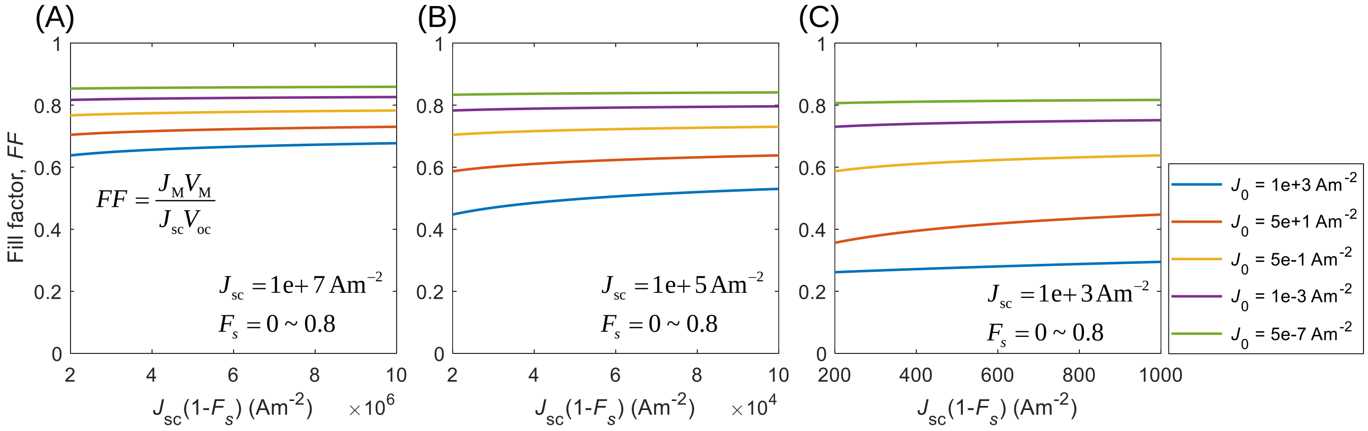

Fig. S2: The effect of the shading fraction F_s on the fill factor FF at given short circuit current density J_{sc} and saturation current density J_0 is analyzed to ensure that FF remains relatively constant regardless of variation in F_s. (A) FF is driven as a function of $J_{sc}(1-F_s)$ where J_{sc} is 1e7 Am^{-2} and F_s is varied between 0 and 0.8. Calculations for five different values of J_0 are provided. The calculation is repeated with (B) J_{sc} = 1e5 Am^{-2} and (C) J_{sc} = 1e3 Am^{-2}. The analysis covers J_{sc} range from 1e3 to 1e7 Am^{-2}, and wide J_0 range from 5e-7 to 1e+3 Am^{-2}. Therefore, we have validated that F_s has minimal influence on FF, irrespective of the J_0 and J_{sc} values in consideration.

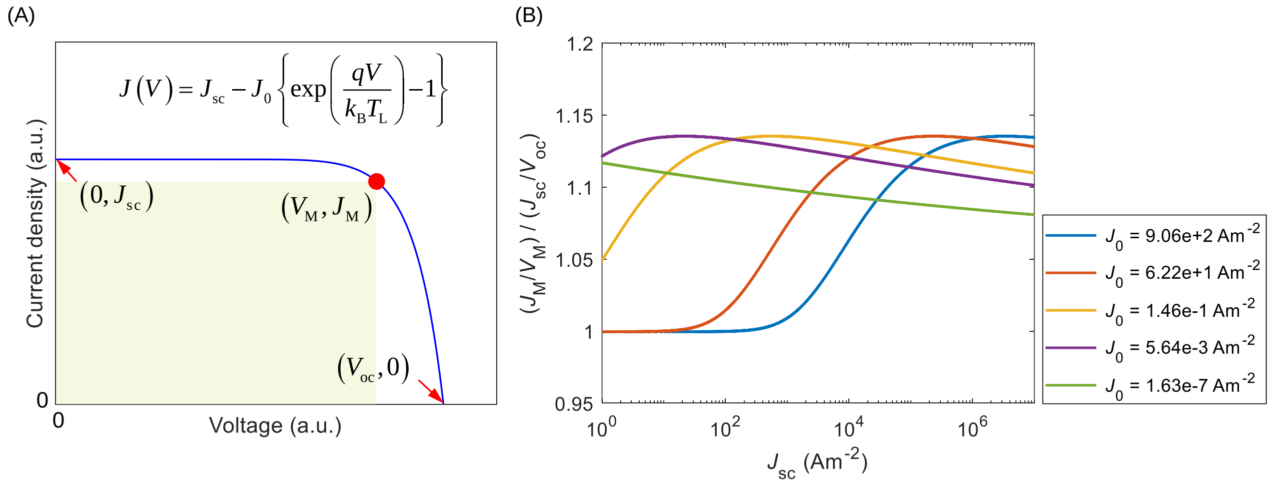

Fig. S3: To confirm the fair similarity between J'_M/V_M and $J_{sc}(1-Fs)/V_{oc,e}$ for the derivation procedure of Eq. (4) in the main text, the ratio of J'_M/V_M to J_{sc}/V_{oc} is calculated across wide ranges of PV cell's performance parameters (i.e., short circuit current density J_{sc} and saturation current density J_0). (A) A J-V characteristic curve of a representative PV cell under illumination is given to illustrate the maximum power point and the two intercepts. (B) The ratio between J_M/V_M and J_{sc}/V_{oc} is calculated as a function of J_{sc} at wide ranges of J_{sc} and J_0, where J_{sc} ranges from 1e1 to 1e7 Am^{-2}, and J_0 ranges from 1.63e-7 to 9.06e+2 Am^{-2}. Since it is observed that $1 \leq (J_M/V_M)/(J_{sc}/V_{oc}) < 1.15$ across wide ranges of J_{sc} and J_0, the approximation of J'_M/V_M to $J_{sc}(1-Fs)/V_{oc,e}$ introduces an error of less than 15%.

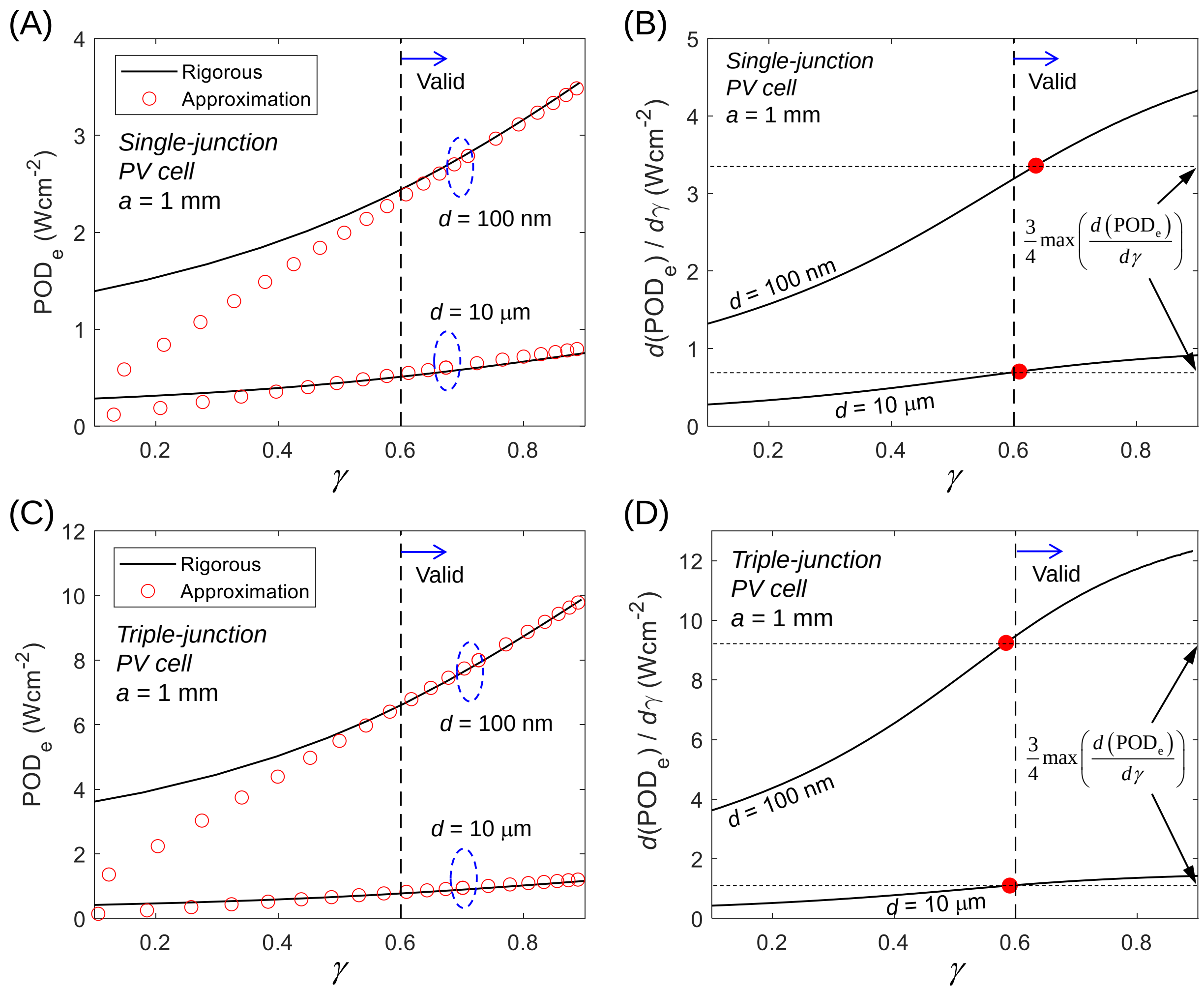

Fig. S4: (A) POD of TPV device utilizing single-junction PV cell as a function of γ. Results obtained with the rigorous and approximative expressions are compared in the near-field (d = 100 nm) and far-field (d = 10 μm) regimes [Fig. 3(A) of the main text repeated]. (B) The validity of the approximate expression as in Eq. (4) in the main text is influenced by the extent to which the linearity between γ and $\mathrm{POD_e}$ changes. To offer a clear boundary for the range of validity, the slope $d(\mathrm{POD_e})/d\gamma$ for the rigorous values in (A) is analyzed as a function of γ. (C) The same analysis as in (A), but with the devices that utilize a triple-junction PV cell [Fig. 3(B) of the main text repeated]. (D) Similar to in (B), the slope $d(\mathrm{POD_e})/d\gamma$ for the rigorous values in (C) is analyzed as a function of γ. Our investigations on the slope in (B) and (D) reveal that approximately 75% of the maximum $d(\mathrm{POD_e})/d\gamma$ (attained when γ approaches 1) serves as the threshold criterion, indicating the extent to which linearity can be maintained. Notably, this value corresponds to a γ of about 0.6, a threshold that remains consistent across varying numbers of junctions and gap sizes. Consequently, we have established $\gamma > 0.6$ as the valid range for our approximative expression.

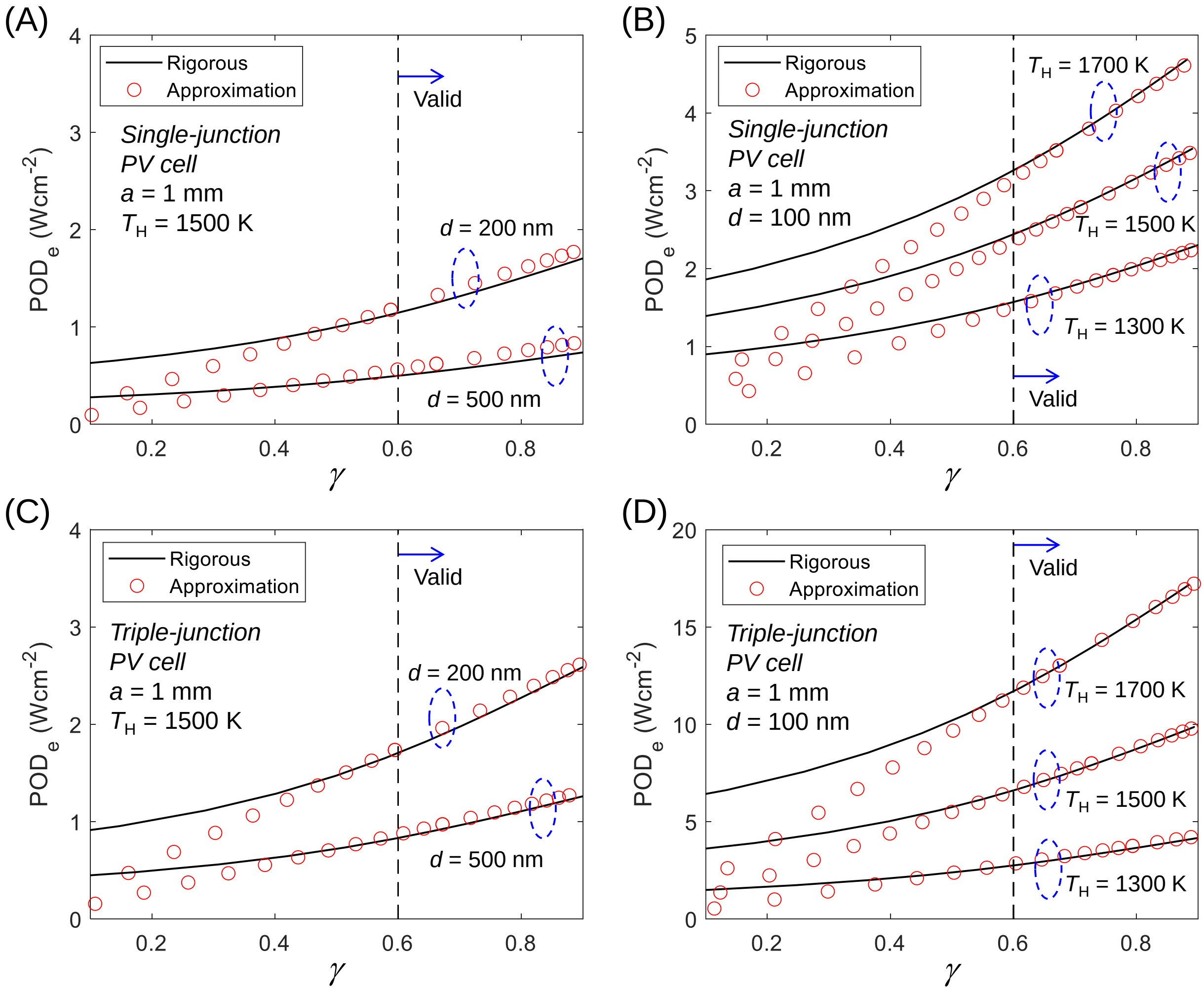

Fig. S5: Comparison of POD calculated with the rigorous and approximative expressions is conducted with vacuum gap sizes (d = 200 and 500 nm), and emitter temperatures (T_{H} = 1300 and 1700 K) different from the parameters that are analyzed within Fig. 3 in the main text. (A) POD of NF-TPV device utilizing single-junction PV cell as a function of γ with T_{H} fixed to 1500 K and d varied as 200 nm and 500 nm. (B) The same analysis as in (A), but with d fixed to 100 nm and T_{H} varied as 1300 K, 1500 K, and 1700K. (C) The same analysis as in (A), but with NF-TPV devices that utilize a triple-junction PV cell. (D) The same analysis as in (B), but with NF-TPV devices that utilize a triple-junction PV cell. The comparison results conclusively demonstrate that our approximative model can precisely estimate $\mathrm{POD_e}$ within the $\gamma > 0.6$, irrespective of the number of junctions, gap sizes, or emitter temperatures.
